# The Calcium Culprit: A Case of Denosumab-induced Hypocalcemia

**DOI:** 10.7759/cureus.4768

**Published:** 2019-05-28

**Authors:** Ricci Kalayanamitra, Ibrahim Yaghnam, Ravi Patel, Andrew Groff, Rohit Jain

**Affiliations:** 1 Emergency Medicine, Penn State Health Milton S. Hershey Medical Center, Hershey, USA; 2 Internal Medicine, Penn State Health Milton S. Hershey Medical Center, Hershey, USA

**Keywords:** denosumab, hypocalcemia, osteoporosis, prolia, denosumab-induced hypocalcemia, drug-induced hypocalcemia, hypophosphatemia

## Abstract

Denosumab is a fully human monoclonal antibody which is used to treat osteoporosis and has been shown to cause hypocalcemia in patients with underlying prostatic and bone malignancies, renal impairment, postmenopausal state, and/or vitamin D deficiency. We present a case of a male patient, with a past medical history negative for the aforementioned conditions, who presented with right shoulder pain and was found to be severely hypocalcemic secondary to denosumab.

## Introduction

Osteoporosis is a bone disease characterized by low bone mass, porous bones, and skeletal fragility, all of which can result in decreased bone strength and an increased risk of fracture. According to the National Osteoporosis Foundation, one in two women and one in four men will break a bone in their lifetime due to osteoporosis. Approximately 44 million people in the USA meet the criteria for osteopenia or osteoporosis. Osteoporosis is responsible for two million fractures per year in the USA, which amount to $19 billion in associated costs annually. This was projected by experts to increase to three million fractures and $25.3 billion in associated costs by 2025 [1]. 

The current first-line management options for osteoporosis are lifestyle changes and bisphosphonate treatment. However, there are limited effective pharmacological options for men with osteoporosis. Denosumab can be considered for these men and for patients who are unresponsive to bisphosphonate treatment [2]. Denosumab is a fully human monoclonal antibody that inhibits the receptor activator of nuclear factor-kB ligand (RANKL) and is commonly used to treat osteoporosis. In 2012, a meta-analysis found denosumab to be significantly better than placebo, zoledronic acid, and pamidronate in reducing the risk of fractures in those with cancer [3]. Within the same year, denosumab was approved by the Food and Drug Administration (FDA) for treating osteoporosis based on data from the ADAMO trial, which displayed efficacy in increasing bone mineral density at the lumbar spine, total hip, femoral neck, hip trochanter, and one-third radius. Denosumab was found to be safe and effective and has the benefits of higher adherence and patient satisfaction rates due to its biannual administration [4-5].

Although denosumab treatment has many benefits, a prospective cohort study published in 2016 found that approximately 25.9% of patients treated with denosumab develop persistent hypocalcemia due to its antiresorptive properties [6]. Despite this being a well-documented side effect of denosumab therapy, there are very few reports of patients presenting with symptomatic hypocalcemia secondary to denosumab use.

## Case presentation

A 74-year-old male with a past medical history of severe osteoporosis, chronic obstructive pulmonary disease (COPD), marginal gastric ulcers status-post sleeve gastrectomy, and Crohn’s disease status-post small bowel resection presented to the emergency department due to an acute onset of right shoulder pain. Vital signs revealed a temperature of 37.2°C, blood pressure of 112/61 mmHg, heart rate of 78, respiratory rate of 16 and oxygen saturation of 92% on 1.5 L by nasal cannula, which he used at baseline. Initial laboratory workup revealed severe hypocalcemia at 4.8 mg/dL and hypophosphatemia at 0.8 mg/dL. Upon further history taking, the patient stated that he receives denosumab injections for his severe osteoporosis. The patient denied muscle cramps, spasms, weakness, seizures, facial twitching, lightheadedness, and psychiatric changes. Of note, he had a history of chronic hypocalcemia with a baseline of 7 mg/dL and reported taking daily calcium and vitamin D supplements. His physical examination was unremarkable except for shoulder pain on passive range of motion. Chvostek and Trousseau signs were both negative. Additional laboratory workup (Table 1) was significant for hyperparathyroidism and normal levels of magnesium, albumin, and vitamin D. His arterial blood gas results (Table 2) revealed respiratory acidosis presumed to be due to COPD.

**Table 1:** Laboratory results from initial workup on presentation to the emergency department.

Test	Result
Serum calcium	4.8 mg/dL
Ionized calcium	0.44 mmol/L
Phosphate	0.8 mg/dL
Magnesium	1.6 mg/dL
Albumin	3.5 g/dL
25-hydroxy vitamin D	42 ng/mL
Parathyroid hormone	354.4 pg/mL

**Table 2:** Arterial blood gas results from initial workup on presentation to the emergency department. pH: potential of hydrogen pCO_2_: partial pressure of carbon dioxide pO_2_: partial pressure of oxygen

Test	Result
pH	7.30
pCO_2 _	64 mmHg
pO_2 _	72 mmHg

The patient was admitted to the intensive care unit, monitored on cardiac telemetry, and started on IV calcium carbonate in addition to his home calcium and vitamin D oral supplements. Endocrinology was consulted and calcium levels were trended until normalization. The patient was ultimately discharged on calcium carbonate 1,250 mg by mouth three times per day, calcium-vitamin D gummies by mouth three times per day, cholecalciferol 1,000 international units by mouth two times per day, and calcitriol 0.5 mcg oral capsule four times per day. His serum calcium level at eight months post-discharge has been maintained at roughly 9.5 mg/dL on this regimen and he has remained asymptomatic throughout.

## Discussion

This case highlights the importance of obtaining a complete medical history, including medication reconciliation, in order to identify drug-induced metabolic abnormalities. Denosumab is injected only once every six months and thus patients often overlook and forget to disclose denosumab as one of their home medications. This is especially true for patients on polypharmacy regimens. There are very few documented reports of patients with denosumab-induced hypocalcemia. The patients in the majority of the previously documented cases of denosumab-induced hypocalcemia have had some form of renal impairment, underlying bone or prostatic malignancies, post-menopausal state, and/or vitamin D deficiency [7-13]. However, the patient in this case developed severe hypocalcemia with a past medical history negative for these conditions. This suggests that denosumab-induced hypocalcemia can occur in patients with good renal function, no underlying malignancy, and normal vitamin D levels. Although the current guidelines recommend monitoring calcium levels only in patients with these underlying conditions and other risk factors predisposing to hypocalcemia, calcium levels should be monitored in all patients receiving denosumab. In addition, according to the FDA guidelines, all patients receiving denosumab should receive daily supplements of 1000 mg of calcium and 400 international units of vitamin D as studies have shown that this prevents episodes of symptomatic hypocalcemia [14]. 

Recent research has shown that denosumab may be more likely to cause hypocalcemia in certain patients due to their baseline bone turnover rate. One study on 85 postmenopausal osteoporotic women being treated with denosumab found that following denosumab injection, the baseline serum bone turnover markers, including total N-terminal propeptide of type 1 procollagen, tartrate-resistance acid phosphatase 5b, and urinary cross-linked N-telopeptide of type 1 collagen, were significantly higher in patients with pre-existing hypocalcemia than in those with normocalcemia [6]. This suggests that denosumab may have a greater impact on the serum calcium levels of osteoporotic patients with higher baseline bone turnover rates than in osteoporotic patients with normal baseline bone turnover rates. Therefore, patients with normal baseline turnover rates can potentially have a decreased chance of developing denosumab-induced hypocalcemia and providers can utilize this information during clinical decision-making.

Although hypocalcemia is a recognized side effect of denosumab and at least one in every four individuals treated with denosumab develops hypocalcemia, it is hypothesized that the majority of patients with this side effect are not seeking care. Severe hypocalcemia usually presents with the common symptoms of perioral paresthesia, carpopedal spasm, tetany, and potentially life-threatening cardiac arrhythmias, but some patients with denosumab-induced hypocalcemia are asymptomatic and therefore are at an increased risk for preventable adverse events because they do not seek medical care due to their lack of symptoms [15-16]. Therefore, we recommend close monitoring of the serum calcium levels in all patients undergoing denosumab therapy and educating these patients on the seemingly harmless symptoms of hypocalcemia that should prompt them to seek medical care. Despite its ability to induce hypocalcemia, denosumab is an effective treatment for osteoporosis and should continue to be cautiously used, especially in those who are unresponsive to lifestyle changes and bisphosphonate treatment.

## Conclusions

Given the occurrence of denosumab-induced hypocalcemia, we suggest that calcium levels should be carefully monitored in all patients on denosumab therapy, regardless of their renal function, lack of an underlying prostate or bone malignancy, post-menopausal state, and vitamin D level. In addition, patients on denosumab therapy should be educated on the symptoms of hypocalcemia so that they may realize the potential severity of their symptoms and seek medical care.
